# Transcriptome responses to heat and cold stress in prepupae of *Trichogramma chilonis*

**DOI:** 10.1002/ece3.7383

**Published:** 2021-03-11

**Authors:** Jiequn Yi, Jianbai Liu, Dunsong Li, Donglei Sun, Jihu Li, Yuxing An, Han Wu

**Affiliations:** ^1^ Guangdong Engineering Research Center for Pesticide and Fertilizer Institute of Bioengineering Guangdong Academy of Sciences Guangzhou China; ^2^ Guangdong Provincial Key Laboratory of High Technology for Plant Protection/Plant Protection Research Institute Guangdong Academy of Agricultural Sciences Guangzhou China

**Keywords:** gene expression, molecular response, thermal stress, transcriptome, *Trichogramma chilonis*

## Abstract

*Trichogramma* is a useful species that is widely applied in biocontrol. Temperature profoundly affects the commercial application of *T. chilonis*. Different developmental transcriptomes of prepupae and pupae of *T. chilonis* under 10, 25, and 40°C were obtained from our previous study. In this study, transcriptomic analysis was further conducted to gain a clear understanding of the molecular changes in the prepupae of *T. chilonis* under different thermal conditions. A total of 37,295 unigenes were identified from 3 libraries of prepupae of *T. chilonis*, 17,293 of which were annotated. Differential expression analysis showed that 408 and 108 differentially expressed genes (DEGs) were identified after heat and cold treatment, respectively. Under heat stress, the pathway of protein processing in endoplasmic reticulum was found to be active. Most of the genes involved in this pathway were annotated as lethal (2) essential for life [*l(2)efl*] and heat shock protein genes (*hsps*), which were both highly upregulated. Nevertheless, most of the genes involved in another significantly enriched pathway of starch and sucrose metabolism were downregulated, including 1 alpha‐glucosidase gene and 2 beta‐glucuronidase genes. Under cold stress, no significantly enriched pathway was found, and the significantly enriched GO terms were related to the interaction with host and immune defenses. Together, these results provide us with a comprehensive view of the molecular mechanisms of *T. chilonis* in response to temperature stresses and will provide new insight into the mass rearing and utilization of *T. chilonis*.

## Graphical abstract

Transcriptomic responses to thermal stresses were characterized in prepupal *Trichogramma chilonis*. Two pathways were significantly enriched after heat treatment which may be important for heat tolerance in *T. chilonis*. A few DEGs were significantly enriched into GO terms related to interactions with host and immune defenses after cold treatment.

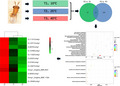

## 1 INTRODUCTION

Temperature is among the most vital abiotic factors that affect the spatial distribution and population abundance of animals (Damos & Savopoulou‐Soultani, 2012; Hoffmann et al., 2003; Wang, Fang, et al., 2014). As ectotherms, insects are exposed to and challenged by temperature stress (Ju et al., 2013; Paaijmans et al., 2013; Yee et al., 2017). Thus, the ability to cope with temperature stress is crucial for the survival of insects (Bartlett et al., 2020; Srithiphaphirom et al., 2019). Insect species have developed various adaptation mechanisms to overcome stressful temperatures during their evolution, which enables species richness and diversification around the world (Dennis et al., 2015; González Tokman et al., 2020; Storey & Storey, 2012).

To date, adaptive responses to temperature stress have been reported, including behavioral, morphological, physiological, and molecular changes in insects (Hoffmann et al., 2003; Musolin & Saulich, 2012; Sejerkilde et al., 2003). Among them, the gene profile changes quickly and plays a versatile role in responses to temperature stress, contributing to physiological resilience (Buckley et al., 2006; Gleason & Burton, 2015). Genes such as the heat shock protein gene (*hsp*) (Ritossa, 1962; Zhao & Jones, 2012) and antifreeze protein (*atf*) (Duman, 1977; Wen et al., 2016) have already been proven to respond to temperature stress, which could maintain the structure and physiological function of insect cells during stress. In recent years, high‐throughput sequencing has been widely used to identify genes and to perform expression profiling (Chen et al., 2019; Wang, He, et al., 2014; Wu et al., 2015). It provides large‐scale genetic information and could broaden our understanding of the underlying mechanisms in insects (Hegde et al., 2003). Transcriptomic responses to temperature stress have been characterized in certain groups of insects, such as *Helicoverpa assulta* (Cha & Lee, 2016), *Nilaparvata lugens* (Huang et al., 2017), and *Drosophila melanogaster* (MacMillan et al., 2016). The adaption mechanism turns out to be a complex progress that involving various molecular changes.

*Trichogramma chilonis* (Hymenoptera: Trichogrammatidae), a tiny egg parasitoid wasp, is widely used in the biological control of numerous lepidopterous pests, including *Chilo* spp., *H. armigera,* and *Pectinophora gassypiella* (Ballal & Singh, 2003; Zhang et al., 2014). It is usually mass‐reared within the temperature range from 25°C to 30°C (Dadmal et al., 2010; Hussain et al., 2013). To meet the great demand for pest control, *T. chilonis* individuals at the prepupal stage were favorable for storage and transport under low temperatures (Yuan et al., 2013). Nevertheless, temperatures beyond the temperature threshold lead to suppression of parasitization, adult emergence, and fertility rates (Haile et al., 2002; Nadeem & Hamed, 2008; Yuan et al., 2012). The ambient temperature could be lower than 0°C in winter and higher than 40°C in summer (Chen et al., 2015; Xiao et al., 2016). Such temperatures could impose various constraints on each process of commercial application of this parasitoid wasp, including mass rearing, storage, and release. Many researchers have noticed and well explored the adverse effects on *Trichogramma* species under high and cold temperatures (Harrison et al., 1985; Nadeem et al., 2009; Schöller & Hassan, 2001). Although our previous studies have confirmed that *hsps* are induced by heat stress, the transcriptomic responses to low‐temperature and high‐temperature stress are still not fully understood (Yi et al., 2018).

Recently, we obtained the transcriptomes of prepupae and pupae of *T. chilonis* exposed to 10, 25, and 40°C for 4 hr and subsequently explored the molecular changes between prepupae and pupae of *T. chilonis* at 25°C (Liu, Yi, et al., 2020). In the present study, we conducted transcriptome profiling to characterize the transcriptomic response to heat and cold stress in the prepupae of *T. chilonis*. Based on the obtained transcriptome data, comparison analysis was further performed to identify differentially expressed genes (DEGs). Quantitative real‐time PCR (qRT‐PCR) was used to examine the thermally responsive DEGs. This study aimed to obtain a comprehensive understanding of the adaptive mechanism of thermal tolerance in *T. chilonis*.

## 2 MATERIALS AND METHODS

### 2.1 Insects and temperature exposure

**FIGURE 1:**
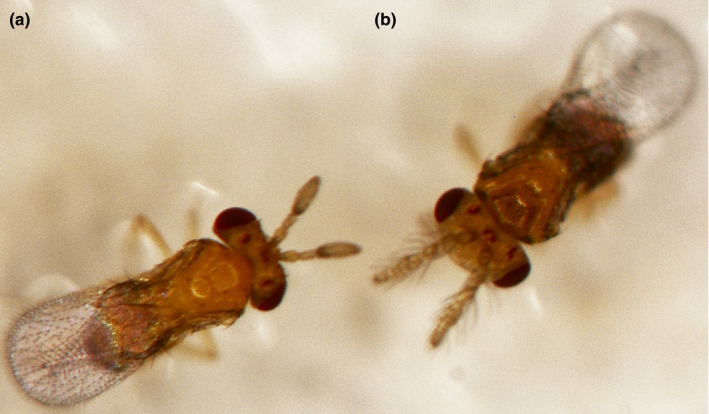
Morphological characteristics of the adult female (a) and male (b) of *Trichogramma chilonis*

The colonies of *T. chilonis* were obtained from the Plant Protection Research Institute, Guangdong Academy of Agricultural Sciences, People's Republic of China and were reared at 25 ± 1°C, 75 ± 5% relative humidity, and a 14 L:10 D photoperiod (Figure 1). The prepupae of *T. chilonis* were confirmed based on our previous studies (Liu, Yi, et al., 2020; Yi et al., 2018), and the corresponding parasitized eggs were exposed to three temperatures: 10°C (T1, cold), 25°C (T2, control), and 40°C (T3, heat) for 4 hr. Then, the parasitized eggs were dissected to collect *T. chilonis* individuals. Individuals were collected when the pulm spots appeared on the body. For qRT‐PCR analysis, each treatment was repeated three times. Each specimen, containing 50 individuals, was immediately frozen in liquid nitrogen and stored at −80°C.

### 2.2 Transcriptome data

We previously obtained the transcriptomes of prepupae and pupae of *T. chilonis* exposed to 10, 25, and 40°C for 4 hr (Liu, Yi, et al., 2020). Using the Illumina HiSeqTM 2500 platform, all these transcriptomes were sequenced at Biomarker Technologies Corporation in Beijing, Beijing, China. The sequencing data have already been available in the NCBI Short Read Archive (SRA) database with the accession number SRP119024. A total of 18,880 unigenes from 6 transcriptomes were previously annotated in 5 public databases, including NR (NCBI, nonredundant protein database), Swiss‐Prot, GO (Gene Ontology), COG (Clusters of Orthologous Groups), and KEGG (Kyoto Encyclopedia of Genes and Genomes). Based on the annotated results of 6 transcriptomes, the unigene annotation information from 3 transcriptomes of prepupae was selected and analyzed.

### 2.3 Differential gene expression analysis and functional annotation

All clean reads from 3 transcriptomes of prepupae were aligned to the unigene library. The results were used to calculate the expression level through RSEM software (http://deweylab.biostat.wisc.edu/RSEM). The relative measure of transcript abundance was fragments per kilobase of transcript per million mapped reads (FPKM). The differential expression analysis of unigenes was conducted between the control (25°C) and the treatments (10°C or 40°C). The false discovery rate (FDR) <0.01 and log_2_fold change (FC) ≥1 were set as the thresholds to screen out the DEGs. GO and KEGG enrichment analyses were applied to determine the significantly enriched GO terms and KEGG terms of DEGs.

### 2.4 Quantitative real‐time PCR analysis

To confirm the results of differential expression analysis, a total of 11 enriched DEGs were selected for qRT‐PCR analysis. Glyceraldehyde‐3‐phosphate dehydrogenase (*gapdh*) was used as the control. Primers were designed by Primer Premier 5.0 and are displayed in Table S1. The total RNA of each group was extracted using TRIzol reagent (Invitrogen according to the manufacturer's protocol. PrimeScriptRT reagent Kit (TaKaRa) was used to synthesize cDNA. The qRT‐PCR was carried out in a LightCycler^®^ 480 Real‐time PCR system (Roche Diagnostics Ltd) using SYBR Green I Master (Roche Diagnostics Ltd.. The results were used to calculate the relative expression levels of chosen genes through the 2^−ΔΔ^ *^Ct^* method.

## 3 RESULTS

### 3.1 Overview of the transcriptome in prepupae of *T. chilonis*

A total of 9.04 Gb bases were obtained from 3 transcriptomes of *T. chilonis* prepupae. The clean reads, Q30, and GC content of each library were over 14,721,157, 92.24%, and 45.95%, respectively, as presented in our previous study (Liu, Yi, et al., 2020). Finally, 37,295 unigenes were identified from 3 libraries (Figure S1).

**FIGURE 2:**
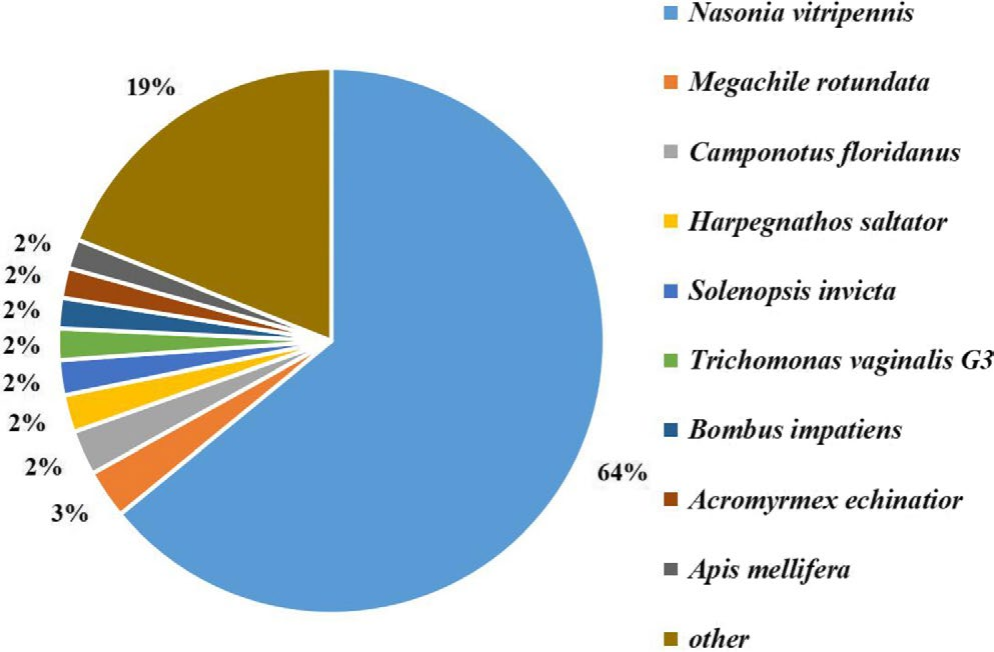
Species distribution of annotated unigenes in the transcriptome of prepupal *T. chilonis*

Among these unigenes, 17,293 unigenes were annotated through 5 public databases (Table 1). According to the NR annotation results, the *T. chilonis* sequences showed high similarity to gene sequences from *Nasonia vitripennis* (64%), *Megachile rotundata* (3%), *Camponotus floridanus* (2%), *Harpegnathos saltator* (2%), *Solenopsis invicta* (2%), *Trichomonas vaginalis G3* (2%), *Bombus impatiens* (2%), *Acromyrmex echinatior* (2%), and *Apis mellifera* (2%) (Figure 2).

**TABLE 1:** Annotation of the transcriptome of prepupal *Trichogramma chilonis*

Database	Number	Ratio (%)
COG	5,692	32.92
GO	8,388	48.51
KEGG	5,181	29.96
Swiss‐Prot	12,862	74.38
NR	17,174	99.31
Total	17,293	100.00

### 3.2 Overview of DEGs under temperature stresses

There were 108 and 408 DEGs in T2 versus T1 and T2 versus T3, respectively (Table 2). These DEGs included 33 up‐ and 75 downregulated genes in T2 versus T1 as well as 212 up‐ and 196 downregulated genes in T2 versus T3. However, only 21 upregulated and 63 downregulated DEGs were annotated in T2 versus T1 (Table S2). The numbers of annotated up‐ and downregulated genes in T2 versus T3 were 142 and 165, respectively. A Venn diagram demonstrates that 41 annotated genes were differentially expressed during both heat and cold exposure (Figure S2). Only 9 DEGs were both upregulated in T2 versus T1 and T2 versus T3, of which 4 DEGs were annotated as lethal(2) essential for life [*l(2)efl*] (CL12907Contig1, CL12768Contig1, CL11322Contig1, CL11101Contig1) (Table S3). Genes such as flavin‐containing monooxygenase FMO GS‐OX‐like 2‐like, BAG domain‐containing protein Samui‐like and cuticular protein LCP family member 2 precursor were also highly expressed in T2 versus T1 and T2 versus T3.

**TABLE 2:** Summary of DEGs during heat and cold stress

DEG set	All DEG	Upregulated (all/annotated)	Downregulated (all/annotated)
T2 vs. T3	408	212/142	196/165
T2 vs. T1	108	33/21	75/63

### 3.3 Functional analysis of DEGs in heat‐stressed *T. chilonis*

Gene Ontology (GO) enrichment analysis for the DEGs was performed in prepupae under heat conditions. Of 408 DEGs, 52 upregulated and 65 downregulated DEGs were annotated in the GO database (Table S2). These DEGs were assigned to 39 GO terms. The most common annotation terms were “catalytic activity” (57 DEGs), “binding” (49 DEGs), “metabolic process” (47 DEGs), and “cellular process” (43 DEGs) (Figure S3). TopGo was subsequently used to infer the primary intersection of DEGs. Enriched GO terms were screened out through the enrichment significance KS < 0.05. The “response to stress” category turns out to be the most enriched GO term, followed by “5'‐flap endonuclease activity” (2 DEGs) and “beta‐glucuronidase activity” (2 DEGs) (Table 3). Most of the DEGs assigned to “response to stress” were *hsps* and *l(2)efls,* which were upregulated in T2 versus T3 (Table S2).

**TABLE 3:** The significantly enriched GO terms in T2 versus T3 and T2 versus T1

Group	GO term	DEGs	Corrected *p*‐value
T2 vs. T1	Interaction with host	2	.00010298
	Cell–cell adhesion	2	.003674098
	Defense response to fungus	2	.00458673
	Defense response to bacterium	2	.012137445
	Positive regulation of DNA binding	1	.049390635
	Host cell part	2	9.25E−05
T2 vs. T3	Response to stress	10	9.58E−10
	5′‐flap endonuclease activity	2	.016526323
	Beta‐glucuronidase activity	2	4.91E−02

**FIGURE 3:**
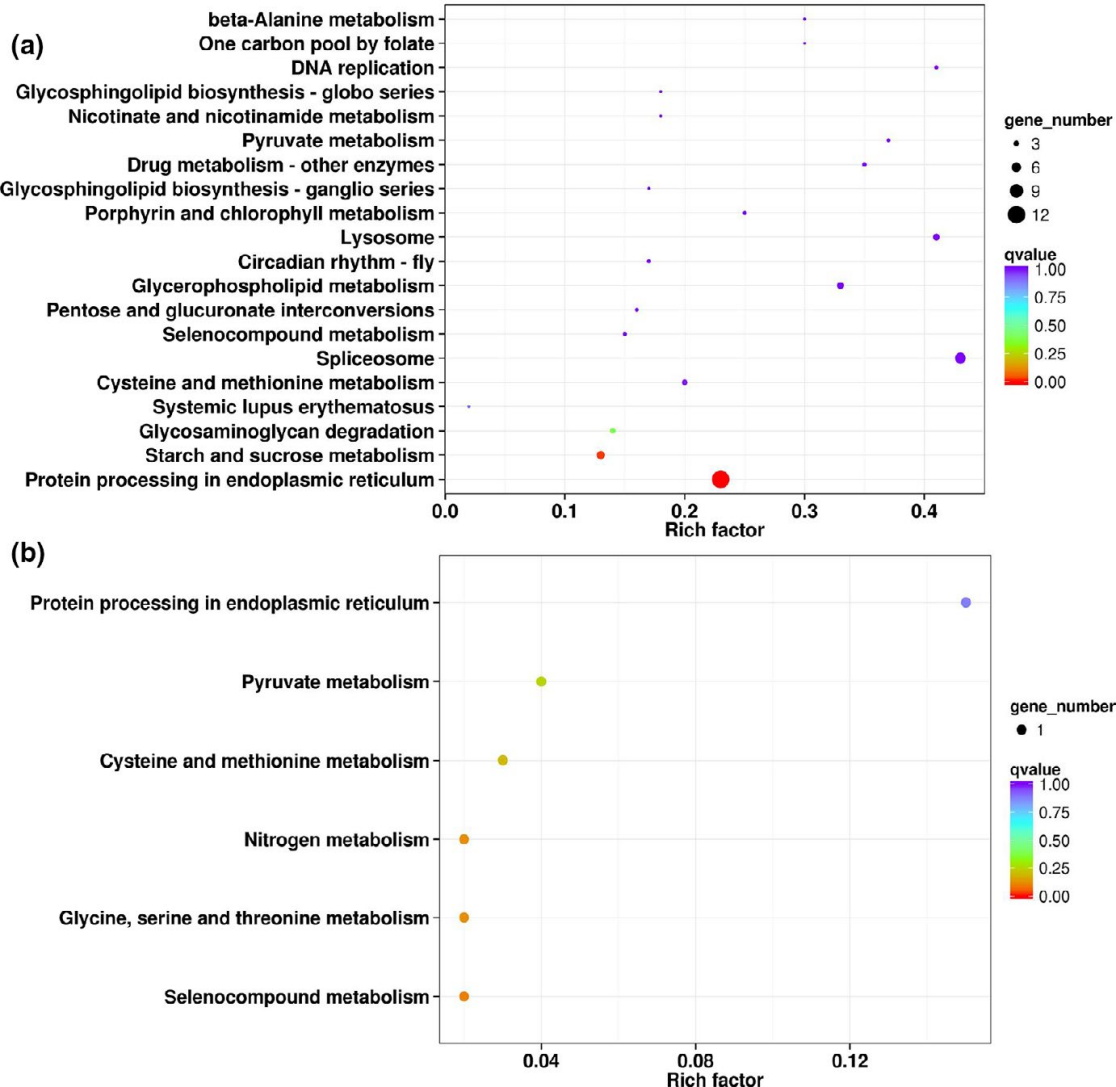
KEGG enrichment analysis of differentially expressed genes (DEGs) in T2 vs. T3 (a) and T2 vs. T1 (b). T1 is low‐temperature group; T2 is control group; T3 is high‐temperature group

KEGG analysis revealed that 80 DEGs in T2 versus T3 could be mapped to 49 pathways (Table S2). Among these terms, “protein processing in endoplasmic reticulum” was the most significantly enriched pathway, containing 12 DEGs (11 upregulated DEGs and 1 downregulated DEG) (Figure 3a). These 11 upregulated DEGs included 5 *l(2)efls* (CL11101Contig1, CL13371Contig1, CL2202Contig1, CL5154Contig1, CL5675Contig1), 4 *hsps* (CL10548Contig1, CL8830Contig1, Group1_Unigene_BMK.11524, Group1_Unigene_BMK.8427), and 2 hypothetical proteins (CL8489Contig1, Group1_Unigene_BMK.11525) (Table 4, Table S2). “Starch and sucrose metabolism” was the second most significantly enriched pathway, containing 4 downregulated DEGs (CL10867Contig1, CL3277Contig1, Group1_Unigene_BMK.17521, Group2_Unigene_BMK.18083) and 1 upregulated DEG (CL1268Contig1) (Table S2).

**TABLE 4:** Differentially expressed HSP family genes and *l(2)efls* under heat and cold stress

Unigene ID	Annotation	T2 vs. T1	T2 vs. T3
CL11101Contig1	Protein lethal(2) essential for life [*Acromyrmex echinatior*]	↑	↑
CL13371Contig1	PREDICTED: protein lethal(2) essential for life‐like [*Nasonia vitripennis*]	–	↑
CL5675Contig1	PREDICTED: protein lethal(2) essential for life‐like [*Nasonia vitripennis*]	–	↑
CL12768Contig1	PREDICTED: protein lethal(2) essential for life‐like [*Nasonia vitripennis*]	↑	↑
CL11322Contig1	PREDICTED: protein lethal(2) essential for life‐like [*Nasonia vitripennis*]	↑	↑
CL2202Contig1	PREDICTED: protein lethal(2) essential for life‐like [*Nasonia vitripennis*]	–	↑
CL5154Contig1	PREDICTED: protein lethal(2) essential for life‐like [*Nasonia vitripennis*]	–	↑
CL12907Contig1	PREDICTED: protein lethal(2) essential for life‐like [*Nasonia vitripennis*]	↑	↑
Group1_Unigene_BMK.8427	heat shock protein 40 [*Pteromalus puparum*]	–	↑
CL8830Contig1	PREDICTED: heat shock protein 83‐like isoform 2 [*Nasonia vitripennis*]	–	↑
Group1_Unigene_BMK.11524	PREDICTED: heat shock 70 kDa protein cognate 4‐like [*Nasonia vitripennis*]	–	↑
CL10548Contig1	PREDICTED: heat shock protein 70 A1‐like [*Nasonia vitripennis*]	–	↑

Note

“↑” means that the unigene is upregulated; “–” means that the unigene is not differentially expressed.

### 3.4 Functional analysis of DEGs in cold‐stressed *T. chilonis*

A total of 6 upregulated and 20 downregulated DEGs were annotated in the GO database. According to the GO annotation, “binding” and “catalytic activity” were the predominant terms in T2 versus T1, containing 15 DEGs and 10 DEGs, respectively (Figure S3). Moreover, 6 significantly enriched GO terms (*p*‐value < .05) were found, namely, “interaction with host” (2 DEGs), “host cell part” (2 DEGs), “cell–cell adhesion” (2 DEGs), “defense response to fungus” (2 DEGs), “defense response to bacterium” (2 DEGs), and “positive regulation of DNA binding” (1 DEG) (Table 3).

Only 3 DEGs in T2 versus T1 could be assigned to 6 pathways (Figure 3b). Two DEGs, namely CL5074Contig1 and CL1586Contig1, were downregulated and involved in metabolic pathways. CL11101Contig1 was the particularly upregulated DEG and participated in “protein processing in endoplasmic reticulum.” This DEG was annotated for *l(2)efl*, which was assigned to the GO term of response to stress by GO enrichment analysis.

### 3.5 Validation of DEG data by qRT‐PCR

**FIGURE 4:**
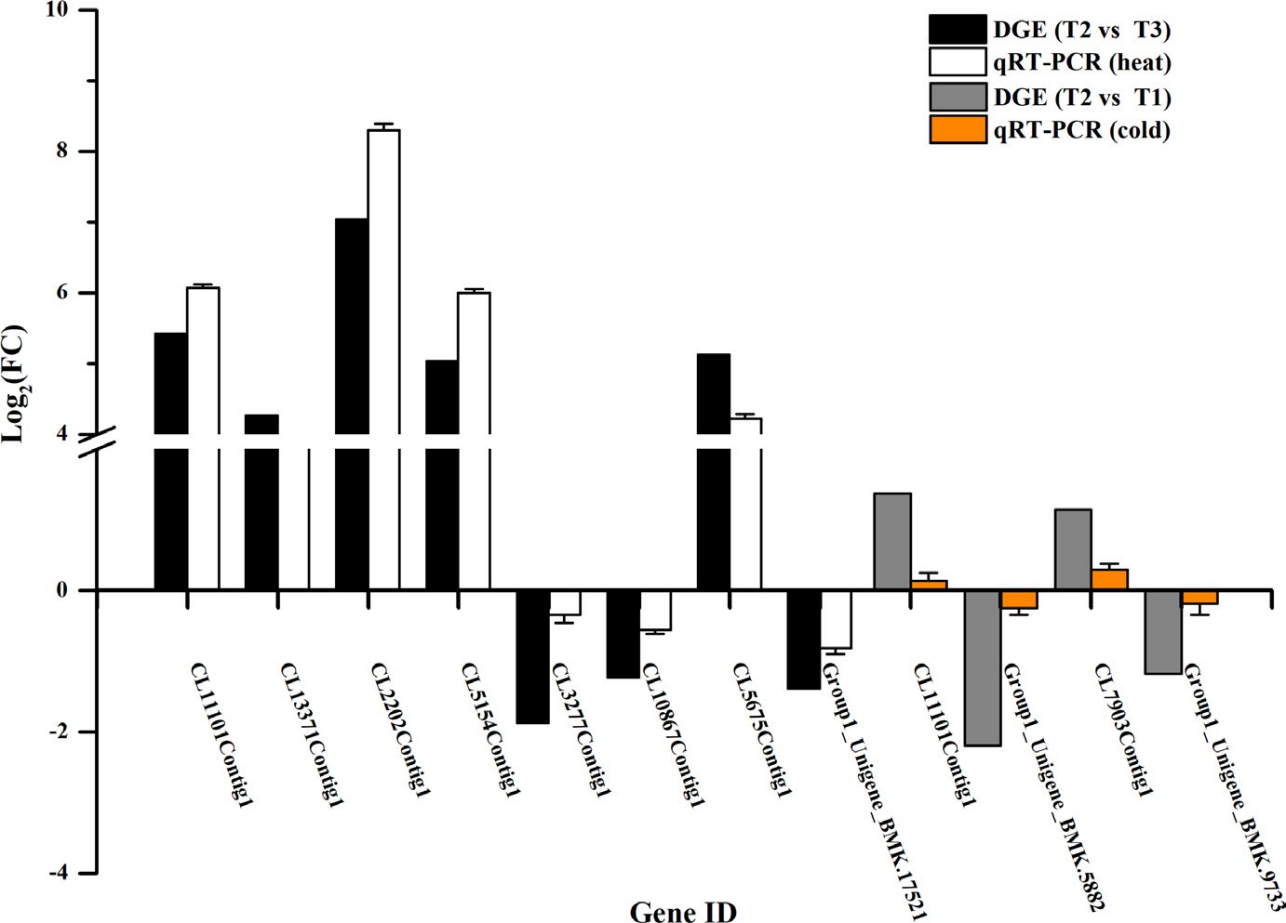
Comparison of fold change in gene expression from DEGs and qRT‐PCR results

The qRT‐PCR results of 11 DEGs are presented in Figure 4. Among these genes, CL11101Contig1 was upregulated after heat and cold stress. During heat stress, the qRT‐PCR results were consistent with the DEG data. During cold stress, although the qRT‐PCR results represented lower expression changes, the changing trend was similar to that of the DEG data.

## 4 DISCUSSION

To date, numerous studies have confirmed that adverse effects appear in *Trichogramma* under temperature stresses (Ayvaz et al., 2008; Harrison et al., 1985; Schöller & Hassan, 2001). However, less research has uncovered the underlying molecular expression pattern in *Trichogramma* species under temperature stresses (Yi et al., 2018). Through our previous work on the developmental transcriptome, a total of 16.88 Gb of clean data were obtained from prepupae and pupae of *T. chilonis* at 10, 25, and 40°C. Of 43,136 unigenes, 18,880 unigenes were annotated (Liu, Yi, et al., 2020). In this study, transcriptomic analysis of *T. chilonis* under temperature stress was performed for the first time. Almost all of the unigenes and annotated genes could be found from 3 transcriptomes of prepupae of *T. chilonis* (37,295 unigenes, 17,293 annotations) (Table 1, Figure S1).

The DEGs obtained from T2 versus T1 and T2 versus T3 are diverse. Only 84 DEGs were annotated during cold exposure, whereas 307 annotated DEGs were obtained during heat exposure (Table 2). In addition, the upregulated DEGs were predominant in T2 versus T3, which is the opposite of the results from T2 versus T1. Different transcriptional profiles were also observed in three rice planthopper species under low and high temperatures in which heat treatment induced more DEGs than cold treatment in *Laodelphax striatellus* (Huang et al., 2017). In fact, certain groups of studies have reported that temperatures up to 40°C impose a heavy strain on *T. chilonis* and that prepupae of *T. chilonis* could survive for a long time at 10°C (Nadeem et al., 2009; Yuan et al., 2013). Together, these results suggested that cold exposure to 10°C for 4 hr was not devastating to the prepupae of *T. chilonis*, which is also demonstrated in the study of the effect of cold storage on the fitness of *Trichogramma* species (Du et al., 2015; Yuan et al., 2013). Although limited annotations and functional classifications were obtained among these DEGs, the involved mechanisms could be explored roughly based on the results of the functional analysis.

After heat and cold treatment, the “protein processing in endoplasmic reticulum” pathway was found in T2 versus T1 and was significantly enriched in T2 versus T3 (Figure 3). It was also found in *Monochamus alternatus* (Li et al., 2019), *Cryptolaemus montrouzieri* (Zhang et al., 2015), and *Cnaphalocrocis medinalis* (Quan et al., 2020) under heat conditions. This pathway favors the correct folding of proteins or degradation of misfolded proteins (Chu et al., 2020; Huang et al., 2018). Here, we found 12 DEGs involved in the “protein processing in endoplasmic reticulum” pathway after heat treatment, most of which were upregulated (Table S2). Among these, 5 genes of *l(2)efl* (CL11101Contig1, CL13371Contig1, CL2202Contig1, CL5154Contig1, CL5675Contig1) and 4 *hsps* (CL10548Contig1, CL8830Contig1, Group1_Unigene_BMK.11524, Group1_Unigene_BMK.8427) were identified and were also enriched in the GO term “response to stress,” suggesting that they may play an important role in the response to heat stress. In many insects, *hsps* are believed to respond to temperature stress (Cheng et al., 2016; Huang et al., 2009; Zhao & Jones, 2012). Although different *hsps* may be induced in different insects or under different treatments, they usually act as molecular chaperones to protect proteins from aggregation and misfolding (Jiang et al., 2012; King & MacRae, 2015; Shi et al., 2013). Another kind of DEGs, *l(2)efls*, are small heat shock‐related genes in response to stress. These genes are considered homologous to small *hsps* in *D. melanogaster* (Chang & Geib, 2018; Kyriakis et al., 1994). They were also found in *Aedes aegypti* (Runtuwene et al., 2020) and *A. mellifera* (Zaluski et al., 2020). In this study, we found 8 different variants in T2 versus T3 and T2 versus T1 (Table 4). They were upregulated not only in T2 versus T3 but also in T2 versus T1, implying that *l(2)efls* are important for thermal adaption in prepupae of *T. chilonis*. These results indicate that genes involved in “protein processing in endoplasmic reticulum” may contribute to the repair or removal of proteins damaged by temperature stresses, which is crucial for thermal tolerance in *T. chilonis*.

The pathway “starch and sucrose metabolism” was another significantly enriched term after heat treatment. Similar to *Glyphodes pyloalis* (Liu et al., 2017), the involved DEGs were partially downregulated. Among these DEGs, 1 alpha‐glucosidase gene (CL3277Contig1) and 2 beta‐glucuronidase genes (Group1_Unigene_BMK.17521 and Group2_Unigene_BMK.18083) were downregulated, indicating repression of carbohydrate metabolism, which may contribute to heat tolerance (Table S2) (Belhadj Slimen et al., 2016; Liu et al., 2017). Interestingly, a trehalase isoform 1 gene (CL1268Contig1) was upregulated after heat treatment, which is used to encode the hydrolyzed enzyme of trehalose (Shukla et al., 2016). In *Galeruca daurica*, this kind of gene was upregulated in summer diapause (Chen et al., 2018). In addition, this gene was also found to play an important role in insect development in Yu's study, in which inhibition of trehalase affected the trehalose and chitin metabolism pathways in *Diaphorina citri* (Hemiptera: Psyllidae) (Yu et al., 2020). Nevertheless, its physiological role in the thermal tolerance of *T. chilonis* still needs to be further characterized.

After cold treatment, 6 pathways were enriched in T2 versus T1 with a corrected *p*‐value > .05 (Figure 3). However, we found 6 significantly enriched GO terms in T2 versus T1, including “host cell part,” “interaction with host,” “cell–cell adhesion,” “defense response to fungus,” “defense response to bacterium,” and “positive regulation of DNA binding” (Table 3). Most of the involved DEGs were downregulated, suggesting that cold stress may alter the fitness and immune defenses of this parasitoid wasp (Iltis et al., 2020). Further analysis revealed that 2 DEGs were hypothetical proteins (Group 1_Unigene_BMK.9173, CL7903Contig1) (Table S2). Interestingly, 1 downregulated DEG was annotated as a heat shock factor protein (Group 1_Unigene_BMK.9733), and no *hsp* was found in T2 versus T1, which was inconsistent with other reports (Kashash et al., 2019; Liu, Han, et al., 2020; Wang et al., 2017). Our previous study revealed that exposure to 10°C could not induce the expression of *hsps* in *T. chilonis* (Yi et al., 2018). This may be an additional molecular evidence that the prepupae of *T. chilonis* exhibited low intensity in response to 4 hr of cold exposure to 10°C.

Temperature stresses also induced a large variety of unigenes that had no clear functional classification. In our study, after cold or heat treatment, cuticular protein LCP family member precursors were positively expressed (Table S3). This has been confirmed in stick insects and *D. melanogaster* (Dennis et al., 2015; MacMillan et al., 2016). During cold, the alteration of cuticles may contribute to avoiding inoculative freezing (Dennis et al., 2015). Two BAG domain‐containing protein Samui‐like genes were also upregulated. They act as heat shock protein cochaperones, regulating the activity and effectiveness of HSPs (Lancaster et al., 2016). Thus, increasing the expression levels of these unigenes may also be beneficial for avoiding damage caused by thermal stresses in the prepupae of *T. chilonis*, as has been reported in *Bombyx mori* (King & MacRae, 2015).

## 5 CONCLUSION

In summary, this study represents the first report on transcriptomic response to thermal stresses in prepupal *T. chilonis*. Transcriptional changes were different under heat and cold stress, with 408 and 108 DEGs in the two treatments, respectively. After heat treatment, a large number of DEGs were significantly enriched in pathways of “protein processing in endoplasmic reticulum” and “protein processing in endoplasmic reticulum,” such as *hsps*, *l(2)efl*s, beta‐glucuronidase genes, and 1 alpha‐glucosidase gene. They may play an important role in the response to heat stress. However, after cold treatment, no significantly enriched pathway was observed. The GO enrichment analysis showed that a few DEGs were enriched in terms related to interactions with host and immune defenses. These results suggested that cold exposure to 10°C for 4 hr may alter the fitness and immune defenses of *T. chilonis* but not devastating prepupae of this parasitoid wasp. Overall, this work provided valuable information for a comprehensive view of the molecular mechanisms of *T. chilonis* in response to temperature stresses.

## CONFLICT OF INTEREST

The authors have declared that they have no competing interests.

## AUTHOR CONTRIBUTION

**Jiequn Yi:** Formal analysis (equal); Funding acquisition (lead); Investigation (lead); Project administration (equal); Validation (equal); Writing‐original draft (lead). **Jianbai Liu:** Formal analysis (equal); Investigation (supporting); Validation (equal). **Dunsong Li:** Investigation (supporting). **Donglei Sun:** Supervision (equal). **Jihu Li:** Supervision (equal). **Yuxing An:** Supervision (equal); Writing‐review & editing (equal). **Han Wu:** Funding acquisition (supporting); Project administration (equal); Writing‐review & editing (equal).

## Data Availability

Transcriptome data are available in the NCBI Short Read Archive (SRA) database with the accession number SRP119024, including the transcriptome T1 (SRX3224507), transcriptome T2 (SRX3224514), and transcriptome T3 (SRX3224519). All supplementary materials are available in Zenodo open data repository (https://zenodo.org/record/4471190#.YBEuUha‐tEY.c).
